# Stimulation of Fengycin-Type Antifungal Lipopeptides in *Bacillus amyloliquefaciens* in the Presence of the Maize Fungal Pathogen *Rhizomucor variabilis*

**DOI:** 10.3389/fmicb.2017.00850

**Published:** 2017-05-15

**Authors:** Parent Zihalirwa Kulimushi, Anthony Argüelles Arias, Laurent Franzil, Sébastien Steels, Marc Ongena

**Affiliations:** ^1^Microbial Processes and Interactions Research Unit, Gembloux Agro-Bio Tech Faculty, University of LiègeGembloux, Belgium; ^2^Laboratory of Biotechnology and Molecular Biology, Faculté des Sciences Agronomiques et Environnement, Université Evangélique en AfriqueBukavu, Congo

**Keywords:** *Bacillus*, fengycin, *Rhizomucor*, signaling, biological control

## Abstract

Most isolates belonging to the *Bacillus amyloliquefaciens* subsp. *plantarum* clade retain the potential to produce a vast array of structurally diverse antimicrobial compounds that largely contribute to their efficacy as biocontrol agents against numerous plant fungal pathogens. In that context, the role of cyclic lipopeptides (CLPs) has been well-documented but still little is known about the impact of interactions with other soil-inhabiting microbes on the expression of these molecules. In this work, we wanted to investigate the antagonistic activity developed by this bacterium against *Rhizomucor variabilis*, a pathogen isolated from diseased maize cobs in Democratic Republic of Congo. Our data show that fengycins are the major compounds involved in the inhibitory activity but also that production of this type of CLP is significantly upregulated when co-cultured with the fungus compared to pure cultures. *B. amyloliquefaciens* is thus able to perceive fungal molecules that are emitted and, as a response, up-regulates the biosynthesis of some specific components of its antimicrobial arsenal.

## Introduction

Biological control involving natural antagonistic microorganisms has emerged as a promising alternative to reduce the use of chemical pesticides in agriculture. Some plant-associated isolates of the *Bacillus amyloliquefaciens* species are particularly efficient biocontrol agents to fight a wide range of plant diseases (Cawoy et al., 2011; Pérez-García et al., 2011). The main mechanisms by which this rhizobacterium provides its protective effect to the plant include the induction of natural defenses in the host via the release of so-called elicitor molecules (Induced Systemic Resistance) (Lugtenberg and Kamilova, 2009; Berendsen et al., 2012); the competition with pathogens for space and nutrients in the same ecological niche; the production of various low-molecular weight antimicrobials, extracellular enzymes, or volatile compounds (VOCs) that can play a short- or long-distance role in direct antagonism of pathogens (Yuan et al., 2012; Ashwini and Srividya, 2013). The biocontrol activity of *B. amyloliquefaciens* thus largely relies on its potential to secrete a range of multifunctional secondary metabolites including cyclic lipopeptides (CLPs). CLPs are synthesized in an mRNA-independent way by modular enzymes [non-ribosomal peptide synthetases (NRPSs) or hybrid polyketide synthases/non-ribosomal peptide synthetases (PKSs-NRPSs); Fischbach and Walsh, 2006; Walsh, 2014]. This leads to a remarkable structural heterogeneity varying from one family to another in the type, number and sequence of amino acid residues as well as in the nature of the peptide cyclization. Within each family, some differences occur in the nature, length, and branching of the fatty acid chain leading to the co-production of various homologes by a single strain (Ongena and Jacques, 2008). The three main CLP families are surfactins, iturins, and fengycins. Surfactins are heptapeptides linked to a fatty acid (length C12–C16) via a cyclic lactone ring structure. Iturins are also heptapeptides bound to a β-amino fatty acid chain, with a length varying from C14 to C17. The iturin group comprises several variants including bacillomycins and mycosubtilins. Fengycins also called plipastatins are lipodecapeptides with an internal lactone ring in the peptidic moiety (Ongena and Jacques, 2008). CLPs globally play important roles in the tritrophic interactions between the *Bacillus* producing strains, the plant and the pathogens (Raaijmakers et al., 2010). Surfactins are powerful biosurfactants, with antiviral activities but low antibacterial or antifungal activities (Peypoux et al., 1999; Tendulkar et al., 2007; Abdallah et al., 2015) while iturins and fengycins mostly display antimicrobial activity against a range of yeasts and filamentous fungi (Thimon et al., 1995; Ongena and Jacques, 2008; Zeriouh et al., 2014). CLPs have also been described for their involvement in root colonization as well as in the systemic stimulation of the host plant immune system leading to ISR. These compounds are thus crucial both for rhizosphere fitness of the producing strains and for their biocontrol potential (Ongena et al., 2005; Romero et al., 2007; Ongena and Jacques, 2008; Raaijmakers et al., 2010; Borriss, 2011; Cawoy et al., 2015; Chowdhury et al., 2015). Our understanding of the cellular regulatory processes driving CLP synthesis in *Bacillus* and other bacterial species like *Pseudomonas* has improved thanks to major advances in comparative genomics and transcriptomics (Raaijmakers et al., 2010). However, very little is still known about the possible impacts of interspecies or interkingdom interactions occurring in the rhizosphere on the production of key biocontrol metabolites such as CLPs.

Maize, the main cereal cultivated in the Democratic Republic of Congo (DR Congo), is gaining importance in terms of annual consumption mainly as flour-derived paste (Tollens, 2003). Despite favorable ecological conditions for cultivation, the production remains low and further decreases due to the depletion of soil nutrients, plant infestation by microbial pathogens and post-harvest degradation of infected seeds (Munyuli, 2002; Mutombo, 2009). Conventional pesticides do not provide a solution for future sustainable agriculture also in countries such as DR Congo. Through this study, we intended to demonstrate the potential of *B. amyloliquefaciens* as efficient biocontrol agent for the control of *Rhizomucor variabilis*, a newly isolated fungal pathogen infesting maize in South Kivu. The objective was to identify the compounds mainly involved in the antifungal activity displayed by the bacterium and to evaluate whether their production may be modulated by the nutritional context and upon interaction between the two microbes.

## Materials and Methods

### Bacterial Strains and Plant Material

The bacterial strains used in this study are listed in **Table 1**. These isolates were selected based on their known capacity to protect plants against fungal phytopathogens. They were routinely cultivated on 868 medium plates (yeast extract 16 g/l, casein peptone 10 g, glucose 20 g/l, agar 17g/l) at 26°C and maintained at 4°C before use. The maize variety used for experiments was Eckavel currently broadcast in Kivu by the Haves Plus program of CGIAR-IITA. Seeds displayed an average 90% germination rate under standard conditions. They were stored in aluminum bags at 4°C, relative humidity (RH) 50%.

**Table 1:** Bacterial strains used in this study and their abilities to produce the three different CLP families.

Strain	Source, reference	Lipopeptide production		
		Surfactin	Iturin	Fengycin
*B. amyloliquefaciens S499*	LabStock (Nihorimbere et al., 2012)	+	+	+
*B. amyloliquefaciens FZB42*	R. Borriss, Humboldt University, Berlin; Germany (Chen et al., 2006)	+	+	+
*B. amyloliquefaciens* GA1	Lab Stock (Arguelles Arias et al., 2009)	+	+	+
*B. amyloliquefaciens* QST713	J. Margolis, Agraquest, USA	+	+	+
*P. polymyxa* PP56	B. McSpadden Gardener, Ohio State University, USA (Cawoy et al., 2015)	-	-	-
*B. subtilis* 98S	B. McSpadden Gardener, Ohio State University, USA (Cawoy et al., 2015)	+	+	+
*B. subtilis* 2504	Lab stock (Ongena et al., 2007)	-	-	+
*B. amyloliquefaciens* CH1	R. Borriss, Humboldt University, Berlin; Germany (Koumoutsi et al., 2004)	-	+	+
*B. amyloliquefaciens* CH2	R. Borriss, Humboldt University, Berlin; Germany (Chen et al., 2006)	-	+	-
*B. amyloliquefaciens* AK3	R. Borriss, Humboldt University, Berlin; Germany (Koumoutsi et al., 2004)	+	-	-
*B. subtilis* BN01	Lab Stock (Cawoy et al., 2015)	-	-	-
*B. subtilis* 168	Lab Stock (Cawoy et al., 2015)	-	-	-

### Fungal Species Identification and Culture Condition

The fungal pathogen was isolated from infected seeds and ears of maize collected in South Kivu (DR Congo, the province extends between 1° 44′13″ and 4° 51′32″ east longitude and between 26° 10′30″ and 29° 14′10″ south longitude). This infected plant material was placed on PDA (potato dextrose agar supplemented with chloramphenicol) plates during 18 days at 25°C. For further purification, the fungus was subcultured five times (every 2 weeks) on the same medium using spores as inoculum. The fungus was then maintained at 25°C by sub-culturing every 2 weeks on PDA medium. Fungal species identification was first based on morphology and microscopy observation (five replicates, independent cultures on PDA plates) and further identified by the laboratory of mycology at the Catholic University of Louvain-La-Neuve in Belgium. It was performed by sequencing the internal transcribed spacer (ITS) gene. Fungal DNA was extracted using EZ-10 Spin Column Fungal Genomic DNA Mini-Preps Kit (Bio Basic, Markham, ON, Canada). Amplification of ITS genes using universal primers ITS1 (TCCGTAGGTGAACCTGCGG)/ITS4 (TCCTCCGCTTATTGATATGC) as well as sequencing was achieved by Macrogen- Europe and DNA sequences were compared against NCBI database using BLAST alignment^1^. In order to evaluate the pathogenicity of the fungus, the pathogen was grown on PDA plates for 4 days at 25°C. The mycelium was collected and placed in peptone water to recover spores. Maize seeds were dipped in a solution of 10^8^ spores/ml for 1 h at 25°C. Four seeds were then placed in Petri dish containing moistened Whatman paper for germination. The assessment of pathogenicity on seedling was evaluated by the incidence of diseases on seed rot, root decay, or reduction in both the number and mass of roots after 25 days.

### Reduction of *Rhizomucor* Infection of Seeds and Seedlings by Bacilli Assessed *In Vitro*

Maize seeds were surface-disinfected by successively soaking in 3% sodium hypochlorite for 3 min and then in 75% ethanol for 2 min. The seeds were then washed extensively with sterile distilled water prior to use. Bacterial inocula were prepared as follows. Strains were cultured on 868 medium at 25°C for 48 h. A fresh colony culture was placed in 9 ml of peptone water (1 g/l Bactopeptone, 9 g/l NaCl, 0.02% Tween 80), and centrifuged at 4000 rpm during 20 min at 4°C. The supernatant was removed and the pellet re-suspended in peptone water and cell concentration was adjusted to 5 × 10^8^ cells/ml (based on the fact that an OD_600_ value of 1 corresponds to 10^8^ cells/ml). For the preparation of fungal inoculum, the pathogens were grown on PDA medium for 10 days. Spores were scrapped from the mycelium in peptone water and centrifuged at 3000 rpm. For pathogen inoculation, the seeds were soaked for 40 min in a fungal suspension (1 ml per seed) at a concentration of 1.4 × 10^7^ spores/ml (Bürker cell chamber counting) and at the same time in a bacterial solution with a concentration of 5 × 10^8^ bacterial cells/ml. Treated seeds were placed in Petri dishes containing watered Whatman paper and incubated at 20°C for 10 days. The efficacy of *Bacillus* to control infection was determined using an arbitrary scale of Disease Reduction Index (DRI) established according to the symptoms observed on the seeds, and on hypocotyl/roots of young seedlings. This scale is as follows: (4) total protection of seed and seedling, no symptoms of pathogen damages, (3) intermediate protection, no damage to the seed or on hypocotyls and roots but few traces of spores on parts of the plant, no clear adverse effect on plant health, (2) serious damages on seeds/seedling due to obvious pathogen growth and infection, and (1) dead seeds and/or total decay of the seedlings.

### Biocontrol Assays in Growth Chambers

These assays were performed on maize plants grown in potting soil (commercial soil DCM-ECOTERA with the following characteristics: 38% dry matter; 20% organic matter; pH 6.5; hydraulic conductivity 1.5 μS/cm; NPK: 7-7.5-8; 1.5 Kg/m^3^) to the third-leaf stage at 25°C and 50% RH. Prior to sowing, sterilized seeds were inoculated with the bacterium as described above while the pathogens were introduced into the growth substrate by mixing 15 g of maize flour inoculated with 4 × 10^8^ spores with 2 kg of potting soil. Both potting soil and maize flour were autoclaved prior to use. Mortality and typical symptoms on surviving plants were used as parameters to evaluate disease reduction according to the following arbitrary scale: (0) wilting and death of the entire plant, (1) 100% of the sheet exhibit symptoms but restricted to few basal leaves of the plant, (2) 50–75% of the sheet displays typical symptoms of the disease, (3) <25% of the leaves show symptoms of the disease, (4) <10% of the leave exhibit the symptoms of the disease, and (5) No visible symptoms on plants.

### Root Colonization and *In Planta* Lipopeptide Production on Perlite-Grown Maize Plantlets

Surface-sterilized maize seeds were soaked for 10 min in the bacterial cell suspension at 10^4^ CFU/ml and placed into a 4-cm-diameter glass tube containing 3 g of perlite substrate and 9 mL of Hoagland solution. All this material was sterilized by autoclave. Plantlets were grown at 25°C with a photoperiod of 16 h. Cultures were stopped after 15 days, and S499 cells colonizing the maize rhizosphere were quantified using 1 g of root samples, after vigorous vortexing in the presence of glass beads. Vegetative cell concentrations were determined by plate counts of serial dilutions in parallel on LB medium, based on typical colony morphology, and on blood agar on the basis of the hemolytic halo typically formed around *Bacillus* colonies after 3 days of incubation at 28°C, as a result of lipopeptide activity. In parallel, lipopeptides were extracted from the rhizosphere by adding 6 mL of acetonitrile/formic acid 0.1% and 2 g of beads to each tube. Tubes were vortexed (5 min) and incubated overnight at 30°C with agitation at 2.6 g. The tubes were centrifuged, and the supernatants were vacuum dried (SpeedVac). Dried residues were suspended in the same solvent and processed as described elsewhere for analysis via UPLC–MS.

### Determination of Lipopeptide Production upon Growth in Root Exudates and Artificial Media

Sterilized seeds were placed in Petri dishes (five seeds per plate) containing moistened Whatman paper and incubated for 14 days in the dark at 22°C. Maize root exudates (MRE) were collected from these seedlings with 5 ml of sterile distilled water for each plate. The solution was used as such after filtration through 0.22 μm. The tomato exudate mimicking (EM) medium was adapted from Nihorimbere et al. (2012) and contained per liter: (NH4)_2_SO_4_ 1 g, CuSO_4_ 1.6 mg, Fe_2_(SO_4_)_3_ 1.2 mg, KCl 0.5 g, KH_2_PO_4_ 0.7 g, MgSO_4_⋅7H_2_O 0.5 g, MnSO_4_ 0.4 mg, MOPS 21 g, Na_2_MoO_4_⋅2H_2_O 4 mg, casamino acids 0.5 g, citrate 2 g, fructose 1.7 g, fumarate 0.5 g, glucose 1 g, malate 0.5 g, maltose 0.2 g, oxalate 2 g, ribose 0.3 g, succinate 1.5 g, yeast extract 1 g, pH 6.5. The LB medium contained tryptone 10 g, yeast extract 5 g, NaCl 10 g, pH 7. Cultures were performed in 24-well plates by inoculating 2 ml of fresh medium (LB, MRE, or EM) with 25 μl of a bacterial cell suspension (OD_600_ = 0.1) corresponding to 1 × 10^7^ CFU/ml. Plates were incubated with continuous shaking for 40 h at 28°C. The supernatants obtained after centrifugation were filtered through a 0.2 μm membrane (Sartorius AG), after pooling the content of three wells (repetitions) for each strain.

### Confrontation Assays on MRE Medium

To conduct the experiments, a small section of mycelium (3 cm) of an 8-day old fungal culture grown on PDA medium was taken and placed on agar-supplemented (15 g/L) MRE medium plate. Afterward, 2 days-old colonies of *Bacillus* grown on 868 medium were collected using a sterile toothpick and deposited on opposite edges of the Petri dish (approximately 3 cm from the piece of mycelium). Antagonistic activity was evaluated by measuring the distance (in mm) between the fungus and the bacterial colony after 3 days growth at 25°C. In order to extract lipopeptides from the inhibition zone on this gelified medium, liquid–solid extraction from 1-cm diameter agar plugs was performed. Briefly, agar samples were mixed with 500 μL of 50% acetonitrile homogenized by vortexing and incubated at 4°C for 4 h. The samples were centrifuged (13,000 *g* for 5 min) and the supernatant was filtered through 0.22 μm before further analysis.

### Lipopeptide Identification and Quantification by UPLC–MS

For the estimation of lipopeptide concentration in all supernatant samples and extracts, the filtrates obtained were analyzed using UPLC–MS with UPLC (Acquity H-class, Waters s.a., Zellik, Belgium) coupled to a single quadrupole mass spectrometer (SQD mass analyzer, Waters) using an C18 column (Acquity UPLC BEH C18 2.1 mm × 50 mm, 1.7 μm). Ten μl was injected and elution was performed with a constant flow rate of 0.6 ml min^-1^ using water/acetonitrile acidified with 0.1% formic acid gradient and compounds were identified/quantified based on their retention times and masses compared to commercial standards (98% purity, Lipofabrik Society, Villeneuve d’Ascq, France) as described by Debois et al. (2014).

### *In Vitro* Fengycin Effects on Growth of *R. variabilis*

Inhibition of the mycelial growth and sporulation of *R. variabilis* by pure fengycin (LipoFabrik, Villeneuve d’Ascq, France) was performed in two ways. Firstly, well-diffusion assay in PDA Petri dish with using different concentration (73, 37, and 18.5 μM diluted in 50% methanol) of pure fengycin was performed in order to evaluate effect on mycelial growth. Briefly, fengycin (100 μL) at different concentrations was put into well and 5-mm diameter plug of mycelium was placed in the middle of the dish. The control treatment consisted of 50% MeOH; after that, plates were incubated at 25°C for 48 h. Mycelium inhibition was determined by measuring the mycelium growth diameter compared with the control. Secondly, in order to estimate minimal inhibitory concentration (MIC) in liquid culture, 96-well microplates containing 150 μL of PDB medium (Potato Dextrose Broth) were inoculated with 30 μL of fungal spore suspension (10^8^ spores/mL) supplemented with different concentration of pure fengycin (18.5; 9.25; 4.5 and 2.5 μM, final concentrations). Plates were then incubated at 25°C for 48 h and the inhibition of pathogen growth was determined by measuring the absorbance at 620 nm (OD 620 nm) using a microplate reader (Beckman). Each experiment was repeated three times.

### Impact of Fungus on Lipopeptide Production

A plug of mycelium from gelified PDA plates was sampled and placed in 10 mL of salt peptone water (5 g/L NaCl, 1 g/L peptone, and 0.2% Tween 80). After centrifugation (10,000 rpm for 5 min) 10 μL of the solution was used to inoculate 10 times diluted MRE medium Petri dishes and incubated for 18 h at 25°C. Afterward, a bacterial colony from an overnight culture on solid 868 medium was picked up and striated on both sides of the already inoculated fungus (2 cm between both microorganisms). Agar plugs were sampled in the inhibition zone after 8, 12, 14, 16, 18, and 24 h of culture for further lipopeptide quantification via UPLC–MS while bacterial cells were scrapped from the producing micro-colonies at the same time-points for gene expression measurements by RT-qPCR.

### RT-qPCR Analysis of CLP Gene Expression

RNA purification was done using a NucleoSpinRNA isolation kit (Macherey-Nagel, Germany) from cells coming from 15 g of agar in the inhibition region. The concentration of RNA was measured using a Thermo Fisher Scientific NanoDrop 2000c UV-Vis spectrophotometer. A quantity of 50 ng RNA was used as a template for RT-qPCR (StepOne Real-Time PCR thermal cycler system, Thermo Fisher Scientific) for fengycin gene expression (primers *fenC*_for: CTGAATCTCTTGCGCCATGT and *fenC*_rev: TGATCTGCTGTGCTCCTTCA) using qPCRBIO SyGreen 1-Step Hi-Rox (NIPPON GENETICS, Germany) according to manufacturer’s protocol. Experiment was performed in triplicate, and negative controls were included for each sample. Gyrase gene expression was used as the reference gene (*gyrA*_for: GAGACGCACTGAAATCGTGA *gyrA*_rev: GCCGGGAGACGTTTAACATA).

### Assays to Test the Effect of Volatile Compounds

The effect of fungal VOCs on CLPs production was evaluated on MRE medium using two-compartments Petri plates (Fernando et al., 2005; Chaves-Lopez et al., 2015). In the first compartment, the bacterium was inoculated by depositing a 10 μl drop of a cell suspension adjusted to an OD of 0.1, whereas in the second compartment, a mycelial disk of freshly pre-cultured *R. variabilis* was deposited. Plates were incubated for 4 days at 25°C and the influence of VOCs on the production of LPs was determined by comparing as described above, the amount of compounds released under these conditions (see above for extraction and quantification by UPLC–MS) with those formed in control plates not inoculated with the pathogen.

### Statistical Analysis

Statistical analysis was carried out with Excel and Statistix 8.0 software. Data from biocontrol assays *in vitro* and in growth chambers were subjected to one-way analysis of variance (ANOVA) and significant differences between the various treatments were detected using (LSD) (least significant difference of means test) at α 0.05.

## Results

### Identification and Characterization of the Fungal Pathogen

Phenotypical characterization of the fungal phytopathogen grown on PDA plate showed a typical cotton candy like texture. At early stage of growth, fungal colonies appeared white before turning grayish to gray after few days of culture. Microscopic observation (Supplementary Figure S1) shows hyaline, broad, non-septate, and branched hyphae. Erected unbranched sporangiophores (up to 12.7 to 16.2 μm wide), which appeared sparingly branched later, was also observable. In addition, sporangia appear globose, yellowish, and up to 70–75 μm in diameter sporangia with ellipsoidal to spherical columellae. Sporangiospores were ellipsoidal and often flattened on one side, measuring 7 to 9 μm × 2 to 4 μm. Sequencing of ITS of the fungal pathogen reveal 100% identity with *R. variabilis* type strain CBS 103.93. Pathogenicity of the fungus was evaluated *in planta* where a reduction of root number of 70.3 ± 15.9% occurred 25 days after infection, similarly a reduction of maize plants mass of 29.2 ± 18.6% was observable compared to uninfected maize plants.

### *B. amyloliquefaciens* S499 Protects Maize Seedlings from Infection by *R. variabilis*

**FIGURE 1:**
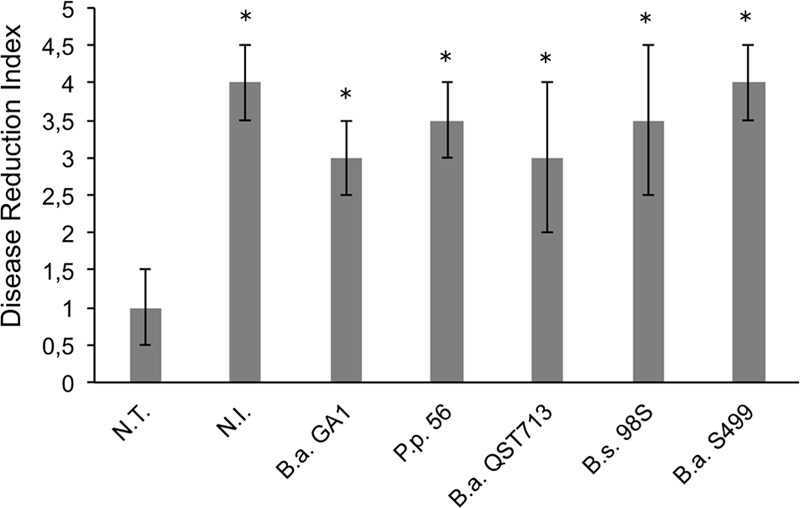
***In planta* control of pathogens on seeds and seedlings.** Efficiency of *Bacillus* strains to reduce infection by *Rhizomucor variabilis* was based on visual assessment after 25 days of incubation [Disease Reduction Index (DRI)]. *N.T*.: seeds are infected with *R. variabilis* and not treated with *Bacillus* strains; *N.I*.: seeds are not infected with the fungus nor treated with *Bacillus* strains. *B.a.* GA1: infected seeds treated with *Bacillus amyloliquefaciens* GA1; P.p 56: infected seeds treated with *Peanebacillus polymyxa* 56; B.a. QST713: infected seeds treated with *B. amyloliquefaciens* QST 713; B.s. 98S: infected seeds treated with *Bacillus subtilis* 98S; and B.a. S499: infected seeds treated with *B. amyloliquefaciens* S499. Averages and standard deviations (error bars) were calculated from four replicates per treatment and the experiment was repeated twice with similar results (*n* = 8). Bars marked with an asterisk differ significantly from the non-treated but infected controls (N.T.) (α < 0.05).

First, we wanted to evaluate *in vitro* the biocontrol potential of different *Bacillus amyloliquefaciens/subtilis* strains on maize seeds and seedlings infected by *R. variabilis*. As shown in **Figure 1**, fungal pathogenicity was confirmed, since all maize seeds infected with *R. variabilis* and not treated with *Bacillus* strains (N.T) were unable to germinated and were fully covered with fungal mycelium (DRI = 1, see Materials and Methods). Interestingly, all tested *Bacillus* strains exhibit good antifungal activity against *R. variabilis* since all of them show DRI comparable to not infected (N.I) plants but *B. amyloliquefaciens* S499 appears to be the most active strain (albeit not significant) since no visual symptoms of fungal disease was observable (DRI = 4) (**Figure 1** and Supplementary Figure S2). Strain S499 was further tested for its ability to control the pathogen and to prevent from take-all and leaf infection on plants grown in infested potting soil to the third-leaf stage. The results showed that bacterial treatment reduced significantly not only the mortality rate (respectively 55 ± 12% and 18 ± 4% for N.T and S499-treated plants) but also leaf infection compared to N.T plants with a high and significant (α < 0.05) reduction of disease severity (respectively 2 ± 0.7 and 4.2 ± 0.9 for N.T and S499-treated plants according to an arbitrary scale of DRI, Supplementary Figure S2).

### Inhibition of *R. variabilis* by S499 on Maize Exudates Correlates with Differential Accumulation of Lipopeptides

**FIGURE 2:**
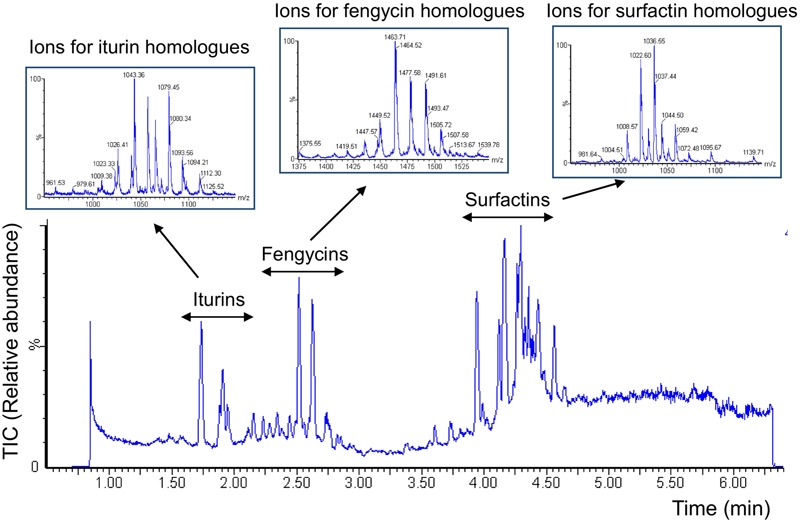
**Diversity of lipopeptides secreted by strain S499 upon growth in maize exudates.** UPLC-ESI-MS chromatogram illustrates the variety of lipopeptides produced by *B. amyloliquefaciens* S499 upon growth in exudates collected from maize seedling as sole nutritional source (MRE medium). Extracted ions typical for surfactins, iturins, and fengycins and representing the various homologes within each lipopeptide family are detailed in boxes.

**FIGURE 3:**
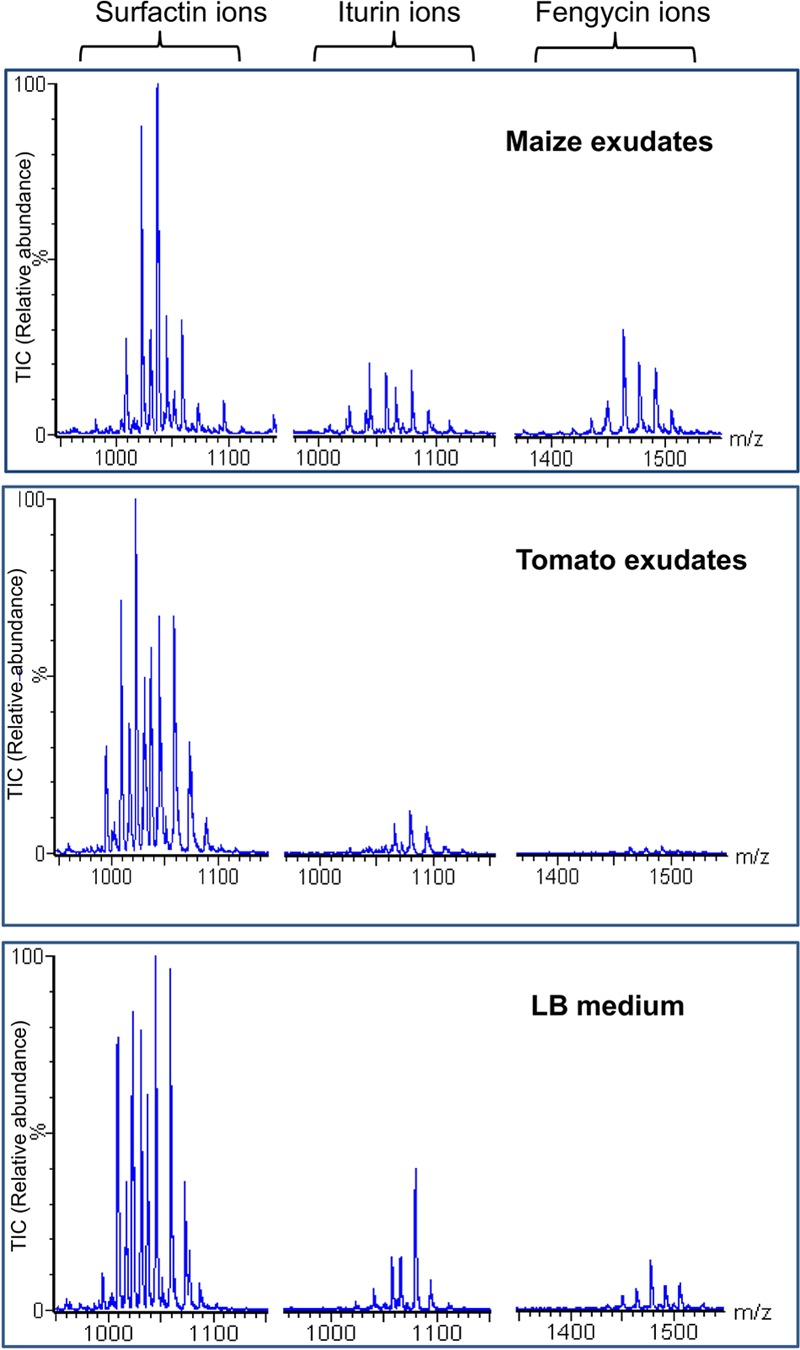
**Efficient production of fengycins by strain S499 in maize exudates.** Extracted ion chromatograms of each lipopeptide family obtained by UPLC-ESI-MS analysis of extracts prepared from supernatants collected after cultivation of strain S499 in the three different media indicated. For each LP family, several peaks are detected which correspond to the various co-produced homologes differing in the length/isomery of the fatty acid tail. Y-axes representing the relative abundance of the ions are linked to the same scale.

Additional experiments were performed on 20 days-old maize plants elevated under gnotobiotic conditions (sterilized perlite as growth substrate) in order to evaluate S499 colonization and possible antibiotic production *in planta*. Data revealed that the bacterium efficiently colonized maize roots to reach populations of 1.1 ± 0.2 CFU/g of root (mean and SD calculated from five plants) and produced surfactins in relevant amounts (2.3 ± 0.4 μg/ml of rhizosphere) as well as some fengycins and iturins but in non-measurable amounts. We used a second approach to appreciate the ability of the *B. amyloliquefaciens* isolate to possibly form secondary metabolites such as lipopeptides under a more realistic nutritional context according to the rhizosphere ecology by growing S499 in exudates collected from maize seedling elevated under gnotobiotic conditions as sole nutritional source (MRE) medium. These naturally produced maize exudates represented an adequate food source as they sustained growth of the S499 strain to an OD_600_ value of approximately 0.8. Lipopeptides of the three different families, i.e., surfactins, fengycins, and iturins (structures in Supplementary Figure S3) were also readily formed in these conditions as detailed in **Figure 2** showing the whole range of typical ions detected by UPLC–MS analysis of the culture supernatant (Debois et al., 2014). Interestingly, these data also revealed high relative amounts of fengycins compared to the blend of lipopeptides released by S499 after growth in other media such as the artificial LB medium or in the EM medium containing C and N sources reflecting root-exuded products from other plant species such as tomato (**Figure 3**).

**FIGURE 4:**
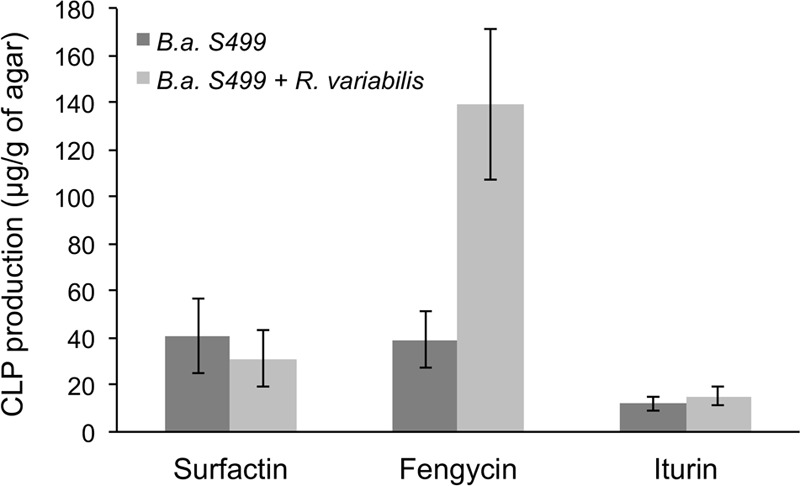
**Determination of lipopeptides accumulating in the inhibition zone upon confrontation of strain S499 with *R. variabilis*.** Production of surfactin, fengycin, and iturin by *B. amyloliquefaciens* S499 evaluated by UPLC–MS in presence (light gray) or absence (dark gray) of *R. variabilis*. Microorganisms were grown together for 4 days at 25°C. Data are means and SD calculated from the analyses of four extracts prepared from independent plates.

Plate confrontation assays were thus performed on gelified MRE medium and a significant inhibition zone (7.2 mm ± 1.7 mm) occurred after 4 days of incubation. Compounds accumulating in that zone were extracted from 1 cm-diameter plugs, and analyzed by UPLC–MS after solid–liquid extraction. Data were compared to those obtained from pure cultures of the bacterial strain performed under the same conditions. Again, lipopeptides were the only compounds detected in significant quantities in those extracts but, interestingly, the amounts of fengycins in the inhibition zone was higher when *B. amyloliquefaciens* S499 was grown in confrontation with the fungus, whereas the presence of the pathogen did not impacted positively or negatively the production of iturins nor surfactins (**Figure 4**). *R. variabilis* did not display any anti-bacterial activity against the *Bacillus* strain (data not shown).

### Fengycins Are the Main Lipopeptides Formed by S499 Restricting Growth of *R. variabilis*

**FIGURE 5:**
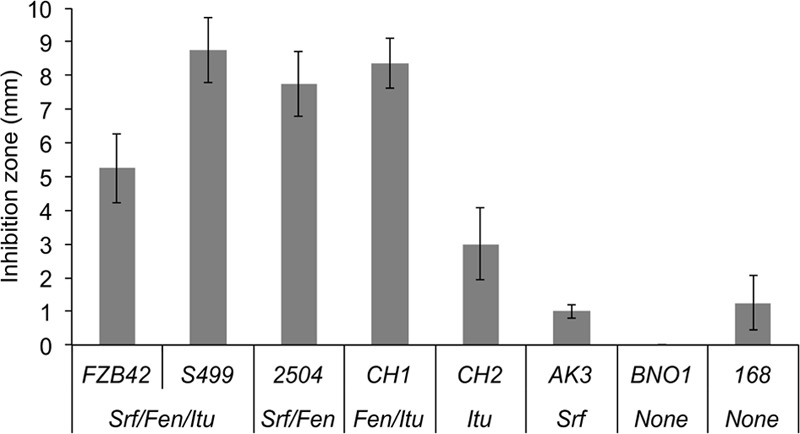
**Fengycins as main ingredients formed by S499 that are active to restrict growth of *R. variabilis*.** Antifungal activity of various Bacillus strains against *R. variabilis* related to the type of CLP family (Srf: surfactins; Fen: fengycins; Itu: iturins; None: no CLPs) that they can produce. Strains unable to produce fengycins do not show any antifungal activity. Presented data are means and SD calculated from four biological repeats.

In order to investigate the role of each lipopeptide family in the control of *R. variabilis*, we first compared the growth inhibition activity on MRE medium of several strains and mutants of *B. amyloliquefaciens/subtilis* producing different combinations of the three families of CLPs. Strains FZB42 and S499 both co-producing iturins, surfactins and fengycins, were able to inhibit the growth of the fungus even if S499 displayed a slightly higher antifungal activity (**Figure 5**). This is in accordance to seedling protection data (**Figure 1**). Strains that do not produce lipopeptides or only very low amounts such as *B. subtilis* BNO1 and *B. subtilis* 168 were not able to inhibit growth of the pathogen highlighting the role of lipopeptides in the antifungal bioactivity. FZB42 derivatives specifically repressed in the synthesis of CLPs (Koumoutsi et al., 2004; Chen et al., 2006; Jourdan et al., 2009; Cawoy et al., 2015) were also included in the experiment. Obtained from the natural strain FZB42, the mutants CH1 and CH2 both producing iturins but not able to form surfactins, only differ from each other in fengycin synthesis which is repressed in CH2 but conserved in CH1. Comparison of antifungal activities of CH1 and CH2 revealed that fengycin production had a strong impact on *R. variabilis* growth indicated a clear involvement of this CLP in fungal inhibition (**Figure 5**). Also surfactin does not play any role in antifungal activity, based on the rather low inhibitory effect of the mutant AK3 also obtained from FZB42, which produces surfactins but is repressed in the synthesis of the two other families. Interestingly, CH1 is more active than the WT strain FZB42, suggesting that surfactins could somehow interfere with fengycin activity. This arises from the fact that the two lipopeptides may interact to probably form a kind of precipitate yielding a “white line” visible around the colonies co-producing all three LP families during antagonism. This was observed for FZB42 and other strains co-producing the three CLP families (Cawoy et al., 2015). Such a phenomenon has also be reported for *Pseudomonas* CLPs involving the lipopeptide so-called WLIP (With Line Inducing Principle) (Rokni-Zadeh et al., 2013). Since *B. amyloliquefaciens* CH2 is still able to slightly inhibit the growth of the phytopathogen, iturins may contribute albeit to a much lower extent, to antifungal activity. The importance of fengycins in the global antifungal effect of *Bacillus* toward *R. variabilis* was also supported by the inhibitory activity of *B. subtilis* 2504, a derivative of the non-active *B. subtilis* 168, in which efficient production of fengycins was restored (Ongena et al., 2007; Nihorimbere et al., 2009).

**FIGURE 6:**
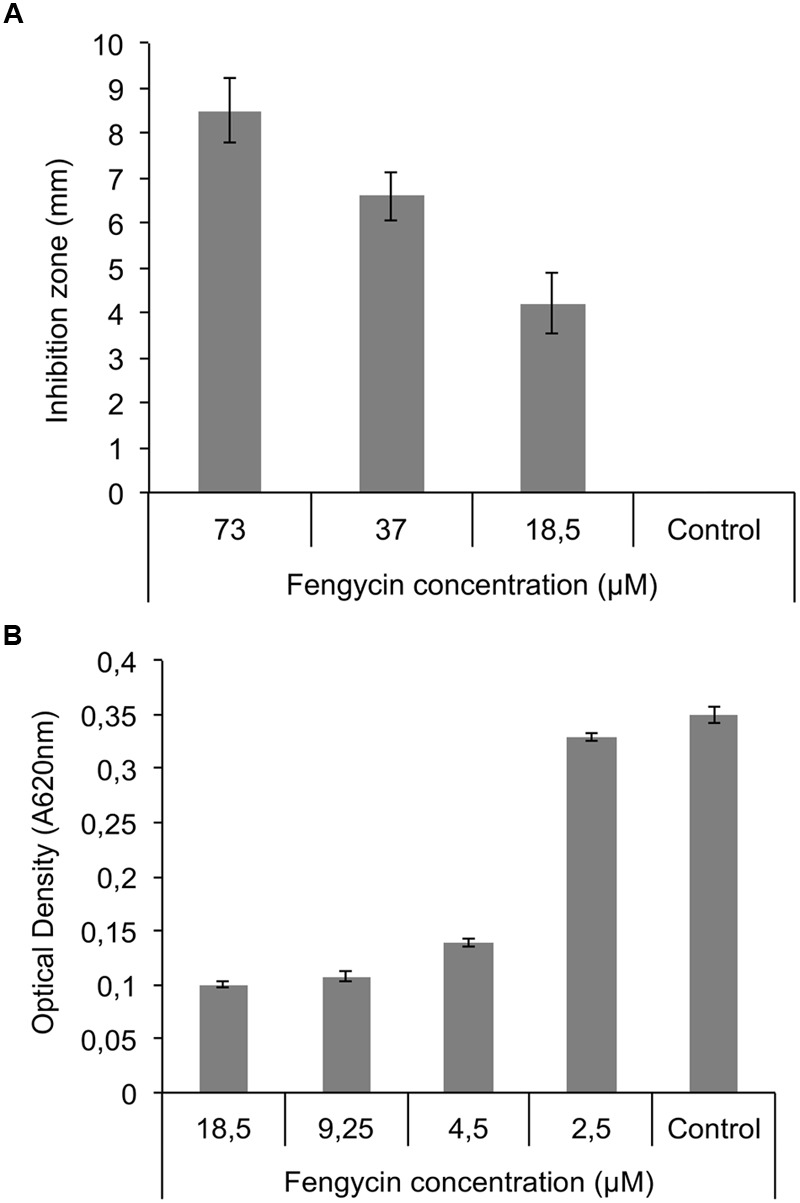
**Fengycin effect on mycelial growth and sporulation of *R. variabilis*. (A)** Effect of pure fengycins on *R. variabilis* growth on PDA plates after 48 h at 25°C in a well-diffusion assay. All tested concentration were able to inhibit growth of fungal mycelium. **(B)** Effect of pure fengycin on sporulation of *R. variabilis* grown in 96-well microplates. Fungal growth was evaluated by measuring OD at 620 nm in presence of different concentrations of fengycins. 4.5 μM is the minimal inhibitory concentration (MIC) capable of inhibiting the growth of the pathogen. Pure fengycins (purity > 99%) were diluted in 50% methanol. Control: 50% methanol. Data are from three independent repeats.

Based on these results, pure fengycins were tested both in a well-diffusion assays for mycelium growth inhibition and in microtiter plates allowing to test the lowest inhibitory concentrations. As shown in **Figure 6A**, fengycin concentrations ranging from 73 to 18.5 μM were inhibitory for mycelium growth on MRE agar plates in a dose response manner. Smaller concentrations from 18.5 to 2.5 μM were also tested in liquid cultures for inhibition of conidia germination and a significant decrease in OD620 was observed by adding pure fengycins at concentrations as low as 4.5 μM, which roughly corresponds to the MIC (**Figure 6B**).

### Fengycin Synthesis by *S499* Is Induced upon Interaction with *R. variabilis*

**FIGURE 7:**
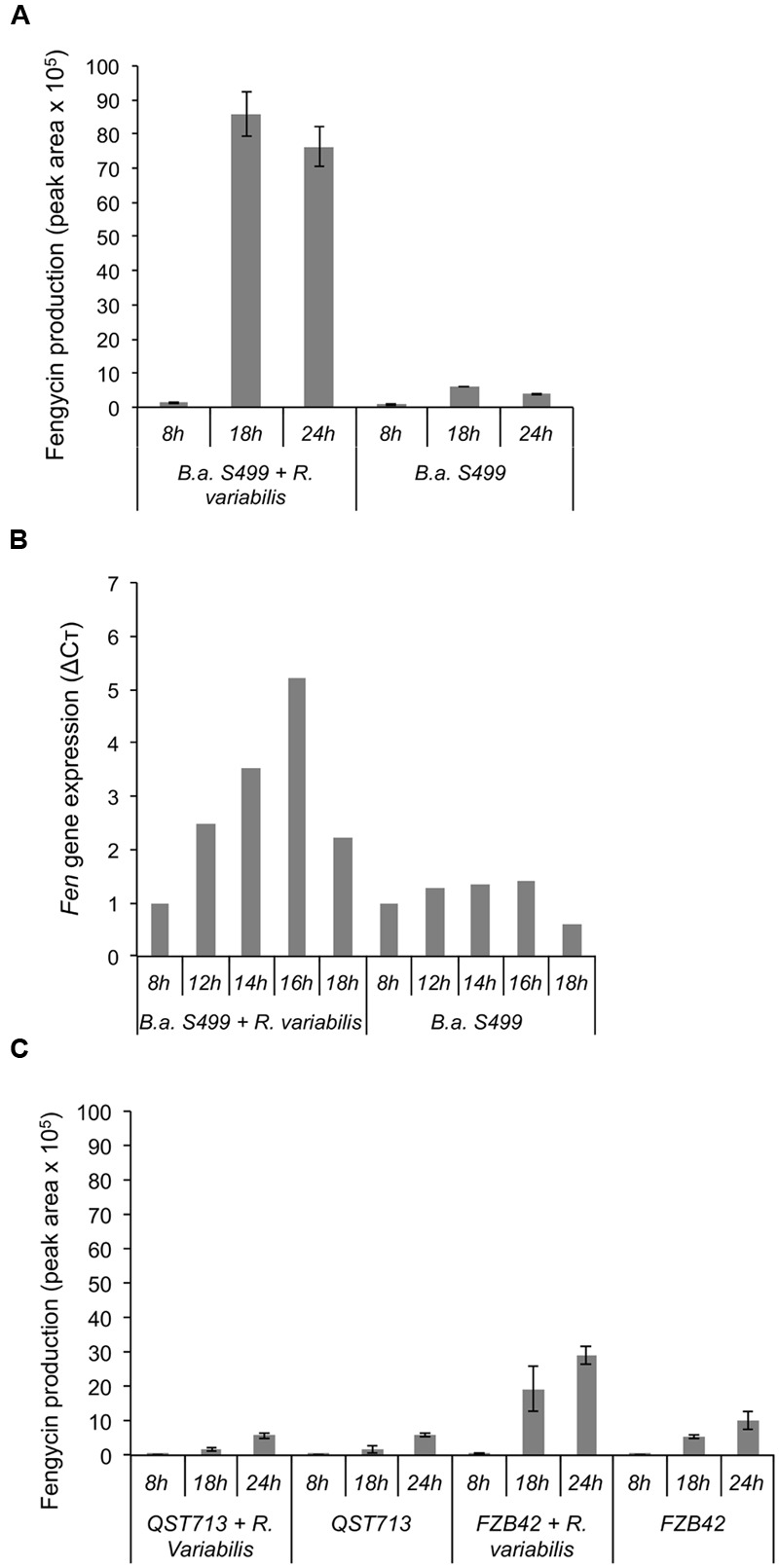
**Impact of the fungus on the production of fengycin and fenC gene expression. (A)** Evaluation of fengycin production by *B. amyloliquefaciens* S499 (*B.a. S499*), using UPLC–MS, in presence (*B.a. S499 + R. variabilis*) or absence (*B.a. S499*) of *R. variabilis* after 8, 18, and 24 h. Positive impact of fungus is clearly visible after 18 and 24 h of co-culture. **(B)** Correlation between *fenC* gene relative expression (ΔCò) and the lipopeptide production. A positive impact on gene expression is clearly visible after 12 h of growth when the fungal pathogen is present compare to culture of *Bacillus* alone. **(C)** Fengycin production by *B. amyloliquefaciens* QST713 and FZB42 in presence (*QST713/FZB42 + R. variabilis*) or absence (*QST713/FZB42*) of *R. variabilis* after 8, 18, and 24 h. For UPLC–MS analysis, data are means and SD from four samples whereas RT-qPCR experiments were performed in duplicate and values at each time-point represent means calculated from two measurements coming from two independent assays.

The dynamic of lipopeptide production by strain S499 in confrontation with *R. variabilis* was further investigated on MRE agar plates with 18 h-delayed inoculation of the bacterium in the vicinity of expanding fungal mycelium. Time-course UPLC–MS profiling of CLPs in the inhibition zone revealed a clear stimulation of fengycin production (up to 10-fold higher amounts) between 8 and 18 h following *Bacillus* inoculation compared to control plates (**Figure 7A**). This increase in fengycin production correlated quite well with an enhanced expression of the corresponding biosynthesis genes as analyzed by RT-qPCR from the producing bacterial colonies. Indeed, evaluation of the expression of the *fenC* gene showed that fengycin synthesis was overexpressed (**Figure 7B**) at 12 h after bacterial inoculation suggesting an early perception of some signal emitted by the fungus. This lipopeptide-inducing molecule emitted by *R. variabilis* most probably corresponds to a soluble compound diffusing in the gelified medium since fengycin stimulation was not observed by growing the bacterium and the fungus under the same conditions but in two-compartments plates commonly used to test the role of volatiles in interspecies interactions (data not shown).

The same experiments were also performed with two other well-described *B. amyloliquefaciens* isolates in order to appreciate the strain specificity regarding such communication between *Bacillus* and *R. variabilis*. It seems that the stimulation of fengycin may also occur for other strains since a similar effect of the fungus, albeit lower in amplitude (5.2-fold increase in fengycin amounts recovered at 18 h post-inoculation), was observed with strain FZB42 but doesn’t seems to apply for all members of this species since no stimulation was detected in the case of strain QST713 (**Figure 7C**).

## Discussion

This work first illustrated the efficacy of some members of the *B. amyloliquefaciens* species and more specifically of the strain S499 at inhibiting *R. variabilis*, a newly isolated endemic fungal pathogen infesting both maize plants in field and ears under post-harvest storage in DR Congo. Our strategy was to further study this antagonistic activity by making both partners interacting upon growth in maize seedling exudates as a nutritional context more representative of natural conditions than the artificial media classically used for that purpose. S499, like other members of the *B. amyloliquefaciens* subsp. *plantarum* taxon is known to produce a panoply of antimicrobial compounds including CLPs of various structural types (Debois et al., 2014; Chowdhury et al., 2015; Molinatto et al., 2016). Our data actually show that this lipopeptidome is readily expressed when the bacterium feeds on these maize exudates as sole nutritional source. Based on the loss of function of specifically repressed mutants and on the activity of purified compounds, it is clear that the fengycin-type lipopeptides mainly contribute to the antifungal potential of strain S499 against *R. variabilis*. This is to our knowledge the first report on fengycin inhibitory activity toward that particular pathogen with a quite low MIC in the μM range. Fengycins were already described for their inhibitory effect on a range of phytopathogenic fungi such as *Podosphaera fusca* (Romero et al., 2007), *Botrytis cinerea* (Touré et al., 2004) and various *Fusarium* species (Hanene et al., 2012; Wang et al., 2015). Fengycins thus acts at low concentrations against several fungi but the molecular mechanisms explaining this antifungal activity are still unclear and may vary according to the pathogen target. In some instances, this was clearly related to the permeabilization of spore/conidia therefore inhibiting germination or alternatively causing hyphal cell perturbation. As revealed by transmission electron microscopy techniques, both phenomena result from membrane damaging by the CLPs (Chitarra et al., 2003; Romero et al., 2007; Etchegaray et al., 2008). This activity most probably relies on the amphiphilic character of these molecules explaining their high affinity for lipid bilayers (Deleu et al., 2008). In support to this mechanism, Wise and colleagues correlated the fengycin activity with the plasma membrane lipid content of the targeted pathogen, by showing that *F. sambucinum, B. cinerea*, and *Pythium sulcatum* were more sensitive to fengycin than *A. solani* having a higher content in ergosterol (Wise et al., 2014) showed that fengycin affected the membrane and cell organs, but also inhibited DNA synthesis in *Rhizopus stolonifer* at low concentrations. The lipopeptide caused hyphal swelling and apoptosis, depolarization and damage of the mitochondrial membrane and the accumulation of ROS in fungal cells (Tang et al., 2014). There are few studies on the effects of fengycins on *R. variabilis*, so it seems that the same mechanisms are at the basis of the strong inhibition of *R. variabilis* mycelial growth and sporulation at low concentrations.

Testing *Bacillus* FZB42 mutants selectively repressed in the synthesis of one or two CLPs revealed that the co-production of surfactin together with fengycin lowered the global antifungal activity. Such a negative effect of mixed CLPs was already observed against *V. dahlia* (Liu et al., 2014). While surfactin and fengycin tested independently readily inhibited spore germination and caused membrane permeabilization, this bioactivity was lost when the two CLPs were applied as a mix. This was also demonstrated on *R. stolonifer*, where surfactin restricted or abolished the inhibitory activity of fengycin on spore germination and hyphal growth (Tao et al., 2011; Liu et al., 2014). This phenomenon may be explained by the stabilizing effect of surfactin on certain lipid bilayers, therefore limiting pore formation in fungal membranes (Grau et al., 1999; Tao et al., 2011). Another possible explanation is that under certain conditions and concentrations, surfactins and fengycins may co-aggregate to form inactive complexes (Cawoy et al., 2015).

Most importantly, we show in this study that fengycin synthesis in *Bacillus* is overexpressed in the presence of *R. variabilis* compared to pure cultures suggesting that the bacterium is able to perceive some molecular trigger(s) released by the pathogen. Many signaling molecules used for communication between microorganisms are VOCs (Fernando et al., 2005; Chaves-Lopez et al., 2015; Giorgio et al., 2015). However, our results strongly suggest that overproduction of fengycins when *B. amyloliquefaciens* S499 is co-cultured with *R. variabilis* is not related to VOCs emitted by the fungus nor to the detection of molecular patterns harbored at the cell surface since the two partners were not in physical contact. Further investigations are still needed in order to identify the triggering molecules released by the fungus. Nevertheless, this CLP stimulation seems to be specific for fengycins since the fungus did not significantly impact the production of iturins and surfactins. Such a modulation of lipopeptide antibiotics and their genes in *Bacillus* upon perception of fungal signals is a rather new concept with a potentially high impact for biological control. Li et al. (2014) have reported that the confrontation of *B. amyloliquefaciens* SQR9 with *Fusarium oxysporum* resulted in an increased production of bacillomycin and fengycin, while when confronted with *Rhizoctonia solani* and *F. solani* there was an increase in the production of surfactin but a decrease in fengycin production. In our recent study, much higher iturin and fengycin production was observed with *B. subtilis* 98S co-cultured with *Pythium* and *Fusarium* compared to controls, but not in the presence of *Botrytis* (Cawoy et al., 2015). So, it appears that a different set of CLPs may be activated in a strain-specific way and depending on the interacting fungal challenger. Still the molecular basis of this phenomenon is fully unknown both regarding fungal perception and downstream signal transduction leading to up-regulated CLP synthesis. We are taking benefit from the fully sequenced genomes of the strains S499 (Molinatto et al., 2016) and FZB42 (Chen et al., 2007) to search for some genetic determinants putatively involved in this regulatory process leading to enhanced expression of the fengycin biosynthesis NRPS machinery upon fungal sensing.

Collectively, our data indicate that the control of *Rhizomucor* infection by S499 observed on seedlings most probably relies on such lipopeptide-dependent direct antagonistic activity of the bacterium toward fungal development. This phenomenon may thus also be involved in the S499-mediated disease reduction observed on more mature plants at the 3–4 leaf stage since both the beneficial and the pathogenic microbes have been inoculated in the potground growth substrate. However, we also show in this work that, upon root colonization of such young maize plants, the bacterium readily forms surfactins while much lower amounts of fengycins and iturins were detected. No other antimicrobials possibly formed by the strain could be detected in these rhizosphere extracts or in exudates. This is in agreement with previous studies on the expression of the S499 antibiome in planta which revealed that CLPs are the main bioactive compounds that are readily secreted by cells colonizing tomato, tobacco, and Arabidopsis roots (Debois et al., 2014). That said, an efficient production of surfactin *in planta* by S499 has been previously correlated with a higher potential for ISR induction compared to other strains belonging to the same subspecies (Jacques et al., 1999; Cawoy et al., 2014). Accordingly, this strain has been reported as strong ISR inducer in a range of plants with surfactin as main elicitor perceived by the host to boost its immunity (Ongena et al., 2007; Cawoy et al., 2014; Debois et al., 2015). Even thought that direct inhibition via the production of fengycins may represent the prime mechanism to explain the protective effect of S499, some stimulation of systemic resistance in the host plant has also to be considered. The recent report of resistance induction in maize by a endophytic and surfactin producing *Bacillus* strain comes in support to that hypothesis.

## Conclusion

It is clear that some environmental factors and plant determinants may influence the production of antibiotics in general and of CLPs in particular in plant beneficial *Bacillus* species (Chowdhury et al., 2015; Debois et al., 2015). This work and others recently conducted in our lab with strain S499 support this statement (Nihorimbere et al., 2012; Pertot et al., 2013; Debois et al., 2015). However, little is still globally known about molecular cues from other plant-associated microbial communities that may impact the expression of this antibiome. In such context, data from this work further illustrate some possible outcomes resulting from interspecies chemical communication. This could be very important for implementing the biocontrol efficacy of *B. amyloliquefaciens* subsp. *plantarum* strains.

## Author Contributions

PZ carried out all the experiments, analyzed the data and wrote the manuscript. AA analyzed the data and wrote the manuscript. LF carried out UPLC-ESI-MS analyses. SS performed RT-qPCR analyses. MO conceived the work, designed the experiments, analyzed the data and wrote/edited the manuscript. All the authors have read the manuscript and agreed to its content.

## Conflict of Interest Statement

The authors declare that the research was conducted in the absence of any commercial or financial relationships that could be construed as a potential conflict of interest.

## Footnotes

1 https://blast.ncbi.nlm.nih.gov/Blast.cgi
