# Supplementary material for: Stimulation of Fengycin-Type Antifungal Lipopeptides in Bacillus amyloliquefaciens in the Presence of the Maize Fungal Pathogen Rhizomucor variabilis
Source: Front Microbiol. 2017 May 15;8:850. doi: 10.3389/fmicb.2017.00850 (PMC5430075; doi:10.3389/fmicb.2017.00850)
Supplement: Supplementary file 1 [file Image_1.pdf]

Supplementary Figure 1

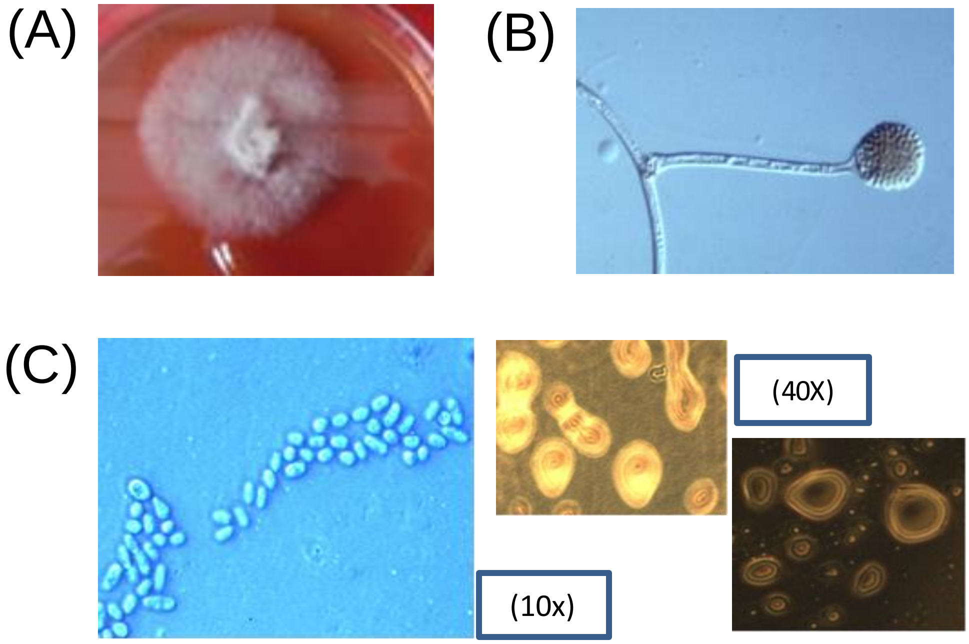

Figure S1: Characterization of the fungal phytopathogen *Rhizomucor variabilis* isolated in this work. A, typical morphology observed for mycelium growing on PDA medium. B, mycelium section and sporangiosphore. C, sporangia.
