# Supplementary material for: Stimulation of Fengycin-Type Antifungal Lipopeptides in Bacillus amyloliquefaciens in the Presence of the Maize Fungal Pathogen Rhizomucor variabilis
Source: Front Microbiol. 2017 May 15;8:850. doi: 10.3389/fmicb.2017.00850 (PMC5430075; doi:10.3389/fmicb.2017.00850)
Supplement: Supplementary file 2 [file Image_2.pdf]

Supplementary Figure 2

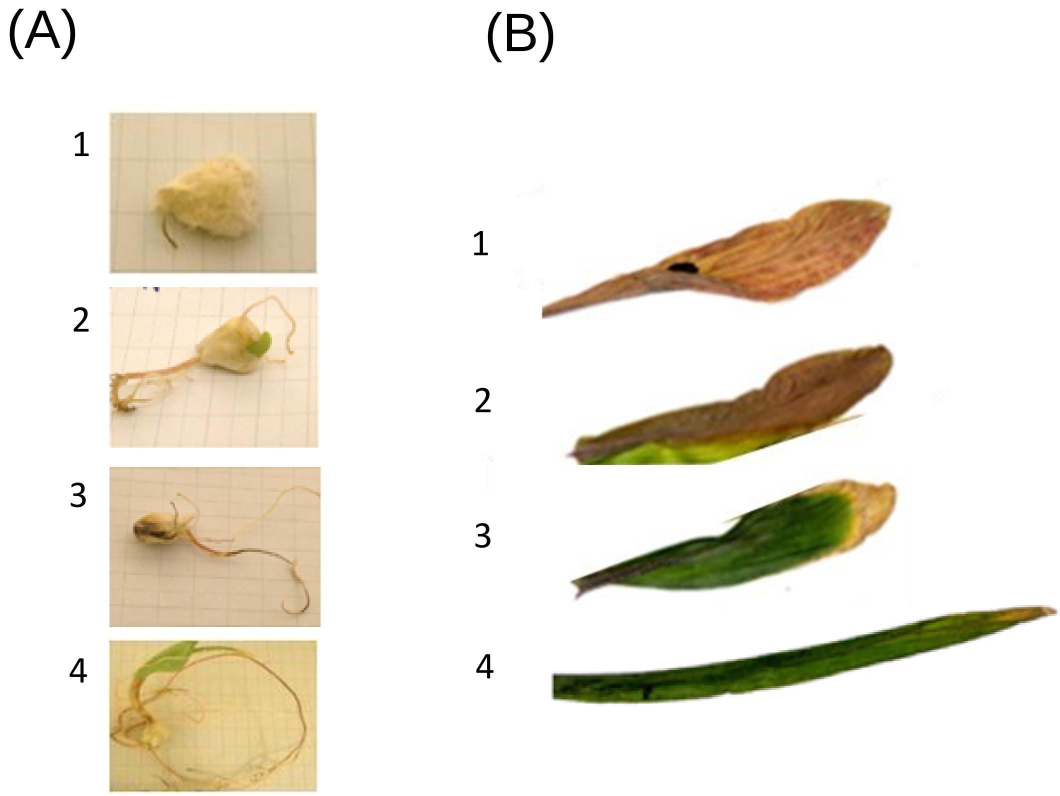

Figure S2: Visualization of biocontrol effect provided by *B. amyloliquefaciens* against *Rhizomucor variabilis*. A, diseases symptoms observed on seeds and seedlings ranging from strong infection (1-2, as observed in disease controls only inoculated with *R. variabilis*) to full protection (4, as observed upon treatment with strain S499). B, leaf infection observed on plants grown in infested potting soil to the third-leaf stage and ranging from severe necrosis on the whole leaf area (1-2, as observed in disease controls only inoculated with *R. variabilis*) to few visible symptoms (3-4, as observed upon treatment with strain S499).
