# Supplementary material for: Stimulation of Fengycin-Type Antifungal Lipopeptides in Bacillus amyloliquefaciens in the Presence of the Maize Fungal Pathogen Rhizomucor variabilis
Source: Front Microbiol. 2017 May 15;8:850. doi: 10.3389/fmicb.2017.00850 (PMC5430075; doi:10.3389/fmicb.2017.00850)
Supplement: Supplementary file 3 [file Image_3.pdf]

Supplementary Figure 3

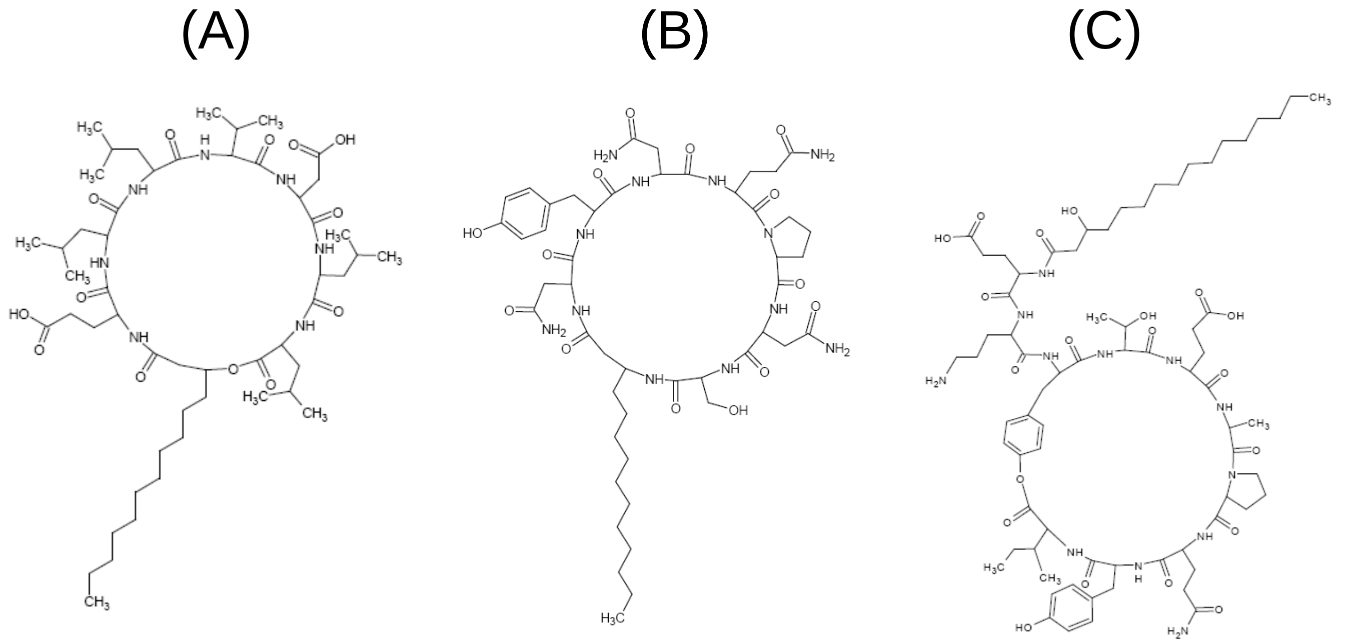

Figure S3: Structures of the main types of lipopeptides formed by members of the *Bacillus subtilis* group. A, Surfactins, heptapeptides with cyclic lactone ring. Several structural variants (amino acid substitutions in the peptide) and various homologues (C12 to C16, linear, iso, anteiso) have been identified. B, Iturins, heptapeptides linked to a β-amino fatty acid. Seven structural variants (Bacillomycins, mycosubtilins, iturins A) and various homologues C_{14} to C_{17} were identified. C, Fengycins, lipodecapeptides with internal lactone ring. Four structural variants and multiple homologues from C_{14} to C_{18} have been reported.
